# The prognostic value of a seven-microRNA classifier as a novel biomarker for the prediction and detection of recurrence in glioma patients

**DOI:** 10.18632/oncotarget.10534

**Published:** 2016-07-11

**Authors:** Wanghao Chen, Qiang Yu, Bo Chen, Xingyu Lu, Qiaoyu Li

**Affiliations:** ^1^ Department of Neurosurgery, People's Hospital Affiliated to Jiangsu University, Zhenjiang, Jiangsu Province, China

**Keywords:** glioma, miRNA, prognosis, LASSO model, recurrence

## Abstract

Glioma is often diagnosed at a later stage, and the high risk of recurrence remains a major challenge. We hypothesized that the microRNA expression profile may serve as a biomarker for the prognosis and prediction of glioblastoma recurrence. We defined microRNAs that were associated with good and poor prognosis in 300 specimens of glioblastoma from the Cancer Genome Atlas. By analyzing microarray gene expression data and clinical information from three random groups, we identified 7 microRNAs that have prognostic and prognostic accuracy: microRNA-124a, microRNA-129, microRNA-139, microRNA-15b, microRNA-21, microRNA-218 and microRNA-7. The differential expression of these miRNAs was verified using an independent set of glioma samples from the Affiliated People's Hospital of Jiangsu University. We used the log-rank test and the Kaplan-Meier method to estimate correlations between the miRNA signature and disease-free survival/overall survival. Using the LASSO model, we observed a uniform significant difference in disease-free survival and overall survival between patients with high-risk and low-risk miRNA signature scores. Furthermore, the prognostic capability of the seven-miRNA signature was demonstrated by receiver operator characteristic curve analysis. A Circos plot was generated to examine the network of genes and pathways predicted to be targeted by the seven-miRNA signature. The seven-miRNA-based classifier should be useful in the stratification and individualized management of patients with glioma.

## INTRODUCTION

Glioblastoma multiforme (GBM), which arises from transformed astrocytes, is the most malignant form of brain neoplasms. GBM is a highly aggressive glioma that is resistant to standard therapeutic interventions, thus its prognosis is often dire.. Currently, GBM has a median survival of 13-16 months post diagnosis, and an overall survival (OS) of 2.5-70 months [1]. While GBM tumors may share similar histological characteristics, their varied molecular characteristics contribute to the vastly different survival rates associated with GBM. Genomic analysis studies have identified molecular signatures which respond to specific treatment regimens [2]. Data from phase III clinical trials suggests that monotherapy with the angiogenesis inhibitor bevacizumab preserves quality of life, reduces corticosteroid use, and improves disease-free survival (DFS); however, bevacizumab monotherapy does not substantially improve OS [3]. Therefore, drugs such as cilengitide are currently used in combination with bevacizumab with the goal of improving OS [4]. A major hurdle in GBM research is identification of prognostic biomarkers that can sensitively predict the clinical outcome of patients.

MicroRNAs (miRNAs) are short 18-25 nucleotide non-coding RNAs that regulate gene expression at the post-transcription level by inhibiting mRNA. Currently, miRNA profiling is an important method that is used to characterize tumors [5]. Bioinformatics tools suggest that miRNAs may regulate more than 60% of human genes including oncogenes, tumor suppressors, and those that impact chemoradioresistance [6]. Moreover, reports suggest that miRNA signatures may be prognostic indicators of GBM thus predict clinical outcome [7-13]. While Cox proportional hazards regression statistics is typically used for modeling covariate analysis associated with patient survival, it is not suitable analyzing high-dimensional microarray data with variable sample size ratio (for example data of less than 10:1). Instead, the least absolute shrinkage and selection operator (LASSO) method has been introduced to eliminate this limitation associated with Cox regression analysis [14]. The LASSO method has been applied broadly to the Cox proportional hazard regression model for survival analysis with high-dimensional data [15]. However, most of the studies that have been performed to identify miRNAs in glioma have used small sample sizes and have not used comprehensive statistical approaches.

The aim of this study was to use advanced statistical methods and an expanded data set to determine a miRNA signature that can proficiently predict DFS and OS in GBM. Using the LASSO Cox regression model and GBM tumor tissues compared with non-tumor brain tissues from patients who underwent surgery, we developed a multi-miRNA-based classifier to predict DFS. We assessed the prognostic accuracy of this classifier using two validation patient groups and confirmed our findings using an independent patient group. Moreover, using bioinformatics analysis, we investigated the functional relevance and ability of the selected miRNAs as potential biomarkers for recurrence and progression. The identification of a validated miRNA signature should be useful in predicting patient outcomes and tailoring treatment regimens for glioma.

## RESULTS

### Identification of miRNAs that are dysregulated in GBM

**Figure 1:**
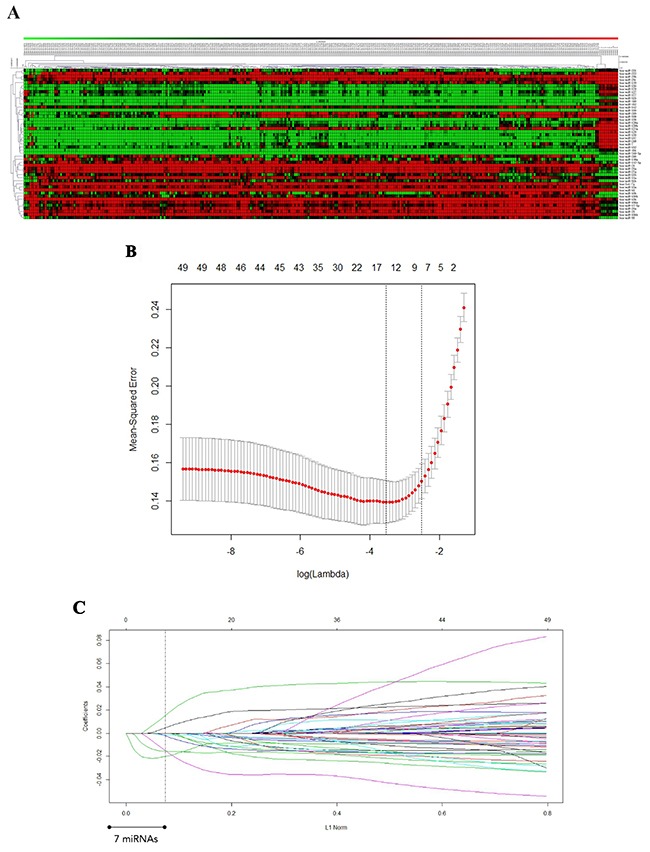
**A.** miRNAs differentially expressed in GBM tissues compared with non-cancerous glial tissues. A heat map of the miRNAs is shown. Green represents miRNAs that are downregulated in GBM, and red represents miRNAs that are upregulated. **B.** Twenty-time cross-validation for tuning parameter selection in the LASSO model. **C.** LASSO coefficient profiles of the 49 GBM -associated miRNAs. A vertical line is drawn at the value chosen by 20-fold cross-validation.

To identify miRNAs that are dysregulated during GBM, we analyzed data from 300 patients from TCGA database who underwent surgical resection for glioma. A training set (89 patients), a validation set (102 patients), and an independent verification set (109 patients) were randomly assigned to GBM patients. A set of 10 unmatched samples from normal non-cancerous tissue was also analyzed for comparison. The distribution of the clinicopathological characteristics of malignant glioma in the three subgroups of GBM patients is shown in Table 1. According to the microarray data, 49 miRNAs were differentially expressed between the 300 GBM samples and 10 matched non-cancerous normal brain tissue samples (fold change ≥ 3.0; false discovery rate 0) (Figure 1A). Of these, 28 miRNAs were upregulated and 21 were downregulated in the 300-sample GMB set (Table 2). Similar dysregulation was observed in the training set, validation set, and independent set (Supplemental Tables 1–3). Hierarchical clustering based on the 49 differentially expressed miRNAs determined that the 300 tumor tissue and 10 normal brain tissue samples could be successfully separated into two discrete groups (p < 0.005); similar groups could be distinguished all three sets.

**Table 1:** Baseline characteristics of patients in three miRNA assessment sets from TCGA

	Training set			Validation set			Independent set		
	Numbers of patients	Low risk	High risk	Numbers of patients	Low risk (%)	High risk (%)	Numbers of patients	Low risk (%)	High risk (%)
**Age**									
≤60 year	40	14(35%)	26(65%)	54	24(44%)	30(56%)	57	29(51%)	28(49%)
>60 year	49	29(59%)	20(41%)	48	20(42%)	28(58%)	52	26(50%)	26(50%)
**Gender**									
Male	50	20(40%)	30(60%)	50	26(52%)	24(48%)	55	19(35%)	36(65%)
Female	39	20(51%)	19(49%)	52	22(42%)	30(58%)	54	36(67%)	18(33%)
**Karnofsky score**									
≤70 score	70	33(47%)	37(53%)	72	34(47%)	38(53%)	84	45(54%)	39(46%)
≥80 score	19	7(37%)	12(63%)	30	12(40%)	18(60%)	25	10(40%)	15(60%)
**Tumorlocation**									
Fronto lobe or temporal lobe	40	19(48%)	21(53%)	48	23(48%)	25(52%)	52	25(48%)	27(52%)
Other lobe*	49	19(39%)	30(61%)	54	25(46%)	29(54%)	57	30(53%)	27(47%)
**Recurrence**									
Yes	69	32(46%)	37(54%)	17	7(41%)	10(59%)	93	41(44%)	53(56%)
No	20	8(40%)	12(60%)	85	41(48%)	44(52%)	16	9(60%)	6(40%)
**MGMT methylight**									
methylated	47	22(47%)	25(53%)	69	38(55%)	31(45%)	78	42(54%)	36(46%)
unmethylated	42	18(43%)	24(57%)	33	10(30%)	23(70%)	31	13(42&)	18(58%)
**IDH1 mutant**									
mutated	11	5(46%)	6(54%)	17	9(53%)	6(47%)	15	9(60%)	6(40%)
unmutated	78	38(49%)	40(51%)	85	36(42%)	49(58%)	94	43(46%)	51(54%)
**Smoking**									
Yes	60	29(48%)	31(52%)	49	22(45%)	27(55%)	77	38(49%)	39(51%)
No	29	11(38%)	18(62%)	53	26(49%)	27(51%)	32	15(47%)	17(53%)
**Family history of cancer†**									
Yes	68	30(44%)	38(56%)	69	30(43%)	39(57%)	88	43(49%)	45(51%)
No	21	10(48%)	11(52%)	33	18(55%)	15(45%)	21	12(57%)	9(43%)

* Other lobe including insular lobe,parietal lobe,cerebellar,lateral ventricles,fourth ventricle, occipital lobe and multiple lobes

† Family history of cancer means any type tumor suffered in any family menbers.

**Table 2:** MiRNAs differentially expressed in GBM tissues compared with non-cancerous glial tissues. The data were derived from 300 GBM patients from TCGA database and 10 controls. (A) Upregulated in GBM; (B) Downregulated in GBM

**MicroRNAs downregulated in Glioblastoma a relative to non-cancer glial tissues**					
**microRNA**	**GBM mean expression**	**Normal mean expression**	**Fold Change**	***p*-value(%)**	**chromosomal location**
hsa-miR-21	40406	1620	24.941	<0.00001	17q23.1
hsa-miR-27a	1801	341	5.283	<0.00001	19p13.12
hsa-miR-15b	14120	3502	4.032	<0.00001	3q26.1
hsa-miR-106b	1404	350	4.01	<0.00001	7q22.1
hsa-miR-93	1152	342	3.369	<0.00001	7q22.1
hsa-miR-23a	13710	3262	4.203	<0.00001	19p13.12
hsa-miR-155	1232	346	3.561	<0.00001	21q21.3
hsa-let-7i	777	299	2.597	<0.00001	12q14.1
hsa-miR-210	1122	340	3.3	<0.00001	11p15.5
hsa-miR-25	1597	348	4.586	0.0009	7q22.1
hsa-miR-130b	997	330	3.025	<0.00001	22q12.21
hsa-miR-92b	887	317	2.802	<0.00001	1q22
hsa-miR-106a	1897	700	2.711	0.0014	Xq26.2
hsa-miR-20a	963	326	2.955	<0.00001	13q31.3
hsa-miR-15a	934	323	2.896	<0.00001	13q14.3
hsa-miR-148a	1168	343	3.405	<0.00001	7p12.5
hsa-miR-16	7587	2959	2.564	0.0018	13q14.3
hsa-miR-17-5p	754	295	2.557	0.00005	3q26.1
hsa-miR-142-3p	1125	340	3.305	<0.00001	17q22
hsa-miR-10b	4076	1014	4.018	<0.00001	2q31.3
hsa-miR-34a	774	299	2.592	<0.00001	1p36.23
**MicroRNAs upregulated in Glioblastoma a relative to non-cancer glial tissues**					
**microRNA**	**GBM mean expression**	**Normal mean expression**	**Fold Change**	***p*-value(%)**	**chromosomal location**
hsa-miR-218	1109	12463	0.089	<0.00001	4p15.31,5q35.1
hsa-miR-129	200	2063	0.097	<0.00001	7q32.1,11p11.2
hsa-miR-491	1687	7271	0.232	0.00006	9p22.3
hsa-miR-132	225	1250	0.18	<0.00001	17p13.3
hsa-miR-137	141	2480	0.057	0.00012	1p21.3
hsa-miR-330	46	163	0.282	0.00009	19q13.32
hsa-miR-139	160	3200	0.05	<0.00001	11q13.4
hsa-miR-124a	60	7444	0.008	<0.00001	8p23.1
hsa-miR-7	173	3270	0.053	0.00074	9q21.32,15q26.1,19p13.3
hsa-miR-769-5p	449	1150	0.39	<0.00001	19q13.32
hsa-miR-323	849	2454	0.346	<0.00001	14q32.31
hsa-miR-128a	474	2928	0.162	<0.00001	2q21.3
hsa-miR-410	751	4289	0.175	0.00023	Xp11.3
hsa-miR-128b	179	1470	0.122	<0.00001	3p22.3
hsa-miR-432	813	2123	0.383	<0.00001	14q32.31
hsa-miR-136	380	1829	0.208	<0.00001	14q32.31
hsa-miR-29c	327	1370	0.239	<0.00001	1q32.2
hsa-miR-338	120	1506	0.08	<0.00001	17q25.3
hsa-miR-342	470	1276	0.368	0.00022	14q32.2
hsa-miR-127	916	4127	0.222	0.00037	14q32.31
hsa-miR-379	771	3293	0.234	<0.00001	14q32.31
hsa-miR-138	102	647	0.158	<0.00001	16q13,3p21.33
hsa-miR-377	361	1281	0.282	<0.00001	14q32.31
hsa-miR-29b	869	2860	0.304	0.00033	7q32.3
hsa-miR-149	141	460	0.307	0.00061	2q37.3
hsa-miR-219	130	1915	0.068	<0.00001	6p21.32,9q32.41
hsa-miR-221	1307	3291	0.397	<0.00001	Xp11.3
hsa-miR-222	236	934	0.253	<0.00001	Xp11.3

† We used these microRNAs in the clustering analysis in Supplementary figure 1.

‡We calculated p values with unpaired class comparison analysis in SAM array Tools 4.0.

### Identification of seven miRNAs significantly associated with DFS in GBM

To further investigate whether the miRNAs are significantly correlated with DFS in GBM, we performed univariate analysis. We used a LASSO Cox regression model to build a prognostic classifier, which selected seven miRNAs from among the 49 miRNAs identified in the training set (Figure 1B and 1C). Among the seven miRNAs, the expression of five miRNAs (miR-124a, miR-129, miR-139, miR-218 and miR-7) was down-regulated in GBM tissues, and the expression of the other two (miR-15b and miR-21) was up-regulated. The association with DFS was verified in all three patient sets (Table 3).

**Table 3:** Univariate association of the 49 differentially expressed miRNAs with DFS in the training, validation, and independent sets. The 7 miRNAs with significant association (P < 0.05) are marked in bold

Parameters	Catergories	Training set		Validation set		Independent set	
		*P* value	HR(95%CI)	*P* value	HR(95%CI)	*P* value	HR(95%CI)
hsa_let_7i	Low(≤ 1.91) *vs.* High expression (> 1.91)	0.07	1.21(0.98-1.49)	0.27	1.09(0.93-1.28)	0.28	1.12(0.91-1.38)
hsa_miR_106a	Low(≤ 0.42) *vs.* High expression (> 0.42)	0.67	1.04(0.86-1.26)	0.76	1.03(0.87-1.21)	0.62	1.06(0.86-1.30)
hsa_miR_106b	Low(≤ 1.93) *vs.* High expression (> 1.93)	0.58	1.21(0.61-2.40)	0.26	1.12(0.92-1.36)	0.34	1.11(0.89-1.39)
hsa_miR_10b	Low(≤ -2.67) *vs.* High expression (> -2.67)	0.58	0.77(0.30-1.97)	0.18	0.94(0.86-1.03)	0.12	0.92(0.83-1.02)
hsa_miR_124a	Low(≤ -2.94) *vs.* High expression (> -2.94)	0.0071	0.75(0.62-0.90)	<0.001	0.81(0.75-0.87)	0.003	0.84(0.76-0.92)
hsa_miR_127	Low(≤ -7.43) *vs.* High expression (> -7.43)	0.48	0.48(0.77-1.13)	0.13	0.87(0.76-1.03)	0.8	1.02(0.87-1.20)
hsa_miR_128a	Low(≤ -6.80) *vs.* High expression (> -6.80)	0.57	0.51(0.74-1.18)	0.1	0.82(0.64-1.04)	0.45	0.91(0.72-1.15)
hsa_miR_128b	Low(≤ -3.54) *vs.* High expression (> -3.54)	0.71	0.70(0.11-4.52)	0.06	0.84(0.69-1.01)	0.4	0.92(0.75-1.12)
hsa_miR_129	Low(≤ -3.27) *vs.* High expression (> -3.27)	0.03	0.04(0.53-0.98)	0.002	0.42(0.31-0.57)	<0.0001	0.51(0.38-0.69)
hsa_miR_130b	Low(≤ 1.78) *vs.* High expression (> 1.78)	0.07	1.19(0.99-1.45)	0.9	1.01(0.88-1.16)	0.39	1.10(0.88-1.38)
hsa_miR_132	Low(≤ 0.43) *vs.* High expression (> 0.43)	0.48	1.04(0.93-1.18)	0.17	1.18(0.93-1.48)	0.65	1.05(0.86-1.28)
hsa_miR_136	Low(≤ -1.37) *vs.* High expression (>1.101)	0.22	0.25(0.03-2.33)	0.11	0.86(0.72-1.03)	0.25	0.83(0.60-1.14)
hsa_miR_137	Low(≤ 1.15) *vs.* High expression (> 1.15)	0.19	0.32(0.06-1.77)	0.26	1.11(0.92-1.34)	0.06	1.25(0.99-1.58)
hsa_miR_138	Low(≤ -4.42) *vs.* High expression (> -4.42)	0.68	0.64(0.08-5.40)	0.57	1.09(0.798-1.51)	0.32	0.90(0.735-1.11)
hsa_miR_139	Low(≤ -1.85) *vs.* High expression (> -1.85)	0.02	1.2(1.03-1.40)	0.014	0.67(0.55-0.83)	0.0004	0.66(0.52-0.83)
hsa_miR_142_3p	Low(≤ -6.22) *vs.* High expression (> -6.22)	0.41	0.54(0.12-2.39)	0.47	0.95(0.84-1.084)	0.83	1.02(0.86-1.21)
hsa_miR_148a	Low(≤ -8.92) *vs.* High expression (> -8.92)	0.13	0.41(0.13-1.29)	0.85	1.01(0.91-1.13)	0.13	1.11(0.97-1.26)
hsa_miR_149	Low(≤ -0.66) *vs.* High expression (> -0.66)	0.54	0.94(0.76-1.16)	0.51	0.96(0.84-1.09)	0.35	0.93(0.80-1.08)
hsa_miR_155	Low(≤ 3.51) *vs.* High expression (> 3.51)	0.62	1.42(0.36-5.62)	0.26	1.10(0.93-1.31)	0.27	1.10(0.93-1.33)
hsa_miR_15a	Low(≤ -0.67) *vs.* High expression (> -0.67)	0.59	0.94(0.73-1.19)	0.7	1.04(0.84-1.30)	0.4	0.91(0.72-1.14)
hsa_miR_15b	Low(≤ 2.2) *vs.* High expression (> 2.2)	0.04	1.25(1.01-1.54)	0.003	1.31(1.09-1.57)	0.003	1.49(1.14-1.95)
hsa_miR_16	Low(≤ 2.55) *vs.* High expression (> 2.55)	0.12	1.29(0.94-1.78)	0.09	1.25(0.97-1.60)	0.73	1.04(0.82-1.32)
hsa_miR_17_5p	Low(≤ -1.51) *vs.* High expression (> -1.51)	0.16	0.86(0.70-1.06)	0.94	0.99(0.84-1.17)	0.54	1.07(0.86-1.33)
hsa_miR_20a	Low(≤ -0.47) *vs.* High expression (> -0.47)	0.63	0.95(0.79-1.16)	0.35	1.08(0.92-1.25)	0.59	1.05(0.87-1.27)
hsa_miR_21	Low(≤ 6.07) *vs.* High expression (> 6.07)	0.002	1.84(1.35-2.50)	0.009	1.38(1.20-1.59)	0.0061	1.37(1.18-1.60)
hsa_miR_210	Low(≤ 6.24) *vs.* High expression (> 6.24)	0.21	1.87(0.70-4.98)	0.17	1.08(0.97-1.21)	0.71	1.03(0.90-1.18)
hsa_miR_218	Low(≤ -2.14) *vs.* High expression (> -2.14)	0.041	0.81(0.66-0.99)	0.0026	0.49(0.36-0.65)	0.013	0.72(0.58-0.93)
hsa_miR_219	Low(≤ -4.04) *vs.* High expression (> -4.04)	0.25	0.67(0.34-1.33)	0.49	1.03(0.95-1.11)	0.35	0.96(0.88-1.05)
hsa_miR_221	Low(≤ 2.24) *vs.* High expression (> 2.24)	0.77	1.25(0.98-1.60)	0.14	1.13(0.96-1.32)	0.26	1.09(0.93-1.30)
hsa_miR_222	Low(≤ -3.49) *vs.* High expression (> -3.49)	0.12	0.71(0.46-1.09)	0.07	1.11(0.99-1.23)	0.53	0.96(0.86-1.08)
hsa_miR_23a	Low(≤ -1.34) *vs.* High expression (> -1.34)	0.57	0.87(0.55-1.40)	0.52	1.06(0.88-1.28)	0.17	1.18(0.93-1.49)
hsa_miR_25	Low(≤ -2.07) *vs.* High expression (> -2.07)	0.42	0.81(0.49-1.35)	0.33	1.12(0.89-1.39)	0.16	1.16(0.94-1.43)
hsa_miR_27a	Low(≤ -0.54) *vs.* High expression (> -0.54)	0.9	0.95(0.39-2.29)	0.65	1.04(0.87-1.26)	0.08	1.23(0.97-1.55)
hsa_miR_29b	Low(≤ 2.45) *vs.* High expression (> 2.45)	0.41	1.28(0.71-2.29)	0.74	0.98(0.84-1.13)	0.61	0.96(0.81-1.13)
hsa_miR_29c	Low(≤ -1.44) *vs.* High expression (> -1.44)	0.29	0.87(0.66-1.13)	0.19	0.89(0.75-1.06)	0.02	0.78(0.64-0.96)
hsa_miR_323	Low(≤ -4.27) *vs.* High expression (> -4.27)	0.51	0.65(0.19-2.25)	0.61	1.06(0.84-1.35)	0.32	1.27(0.79-2.06)
hsa_miR_330	Low(≤ -3.15) *vs.* High expression (> -3.15)	0.18	0.74(0.46-1.16)	0.74	1.05(0.78-1.41)	0.76	0.95(0.67-1.34)
hsa_miR_338	Low(≤ -1.24) *vs.* High expression (> -1.24)	0.6	0.88(0.56-1.40)	0.12	1.09(0.98-1.22)	0.4	1.05(0.94-1.16)
hsa_miR_342	Low(≤ 1.09) *vs.* High expression (> 1.09)	0.67	0.90(0.54-1.48)	0.51	0.91(0.69-1.20)	0.32	0.86(0.64-1.16)
hsa_miR_34a	Low(≤ 4.32) *vs.* High expression (> 4.32)	0.48	1.54(0.46-5.12)	0.08	1.11(0.99-1.25)	0.19	1.10(0.95-1.28)
hsa_miR_377	Low(≤ -1.74) *vs.* High expression (> -1.74)	0.12	0.18(0.02-1.55)	0.14	0.89(0.76-1.04)	0.37	0.92(0.76-1.11)
hsa_miR_379	Low(≤ 2.44) *vs.* High expression (> 2.44)	0.29	1.28(0.82-1.99)	0.68	0.98(0.88-1.09)	0.88	1.02(0.82-1.25)
hsa_miR_410	Low(≤ -6.92) *vs.* High expression (> -6.92)	0.31	0.50(0.14-1.85)	0.37	0.91(0.73-1.12)	0.11	1.22(0.95-1.57)
hsa_miR_432	Low(≤ -0.52) *vs.* High expression (> -0.52)	0.82	0.95(0.61-1.48)	0.14	0.75(0.51-1.10)	0.11	0.72(0.47-1.08)
hsa_miR_491	Low(≤ -0.58) *vs.* High expression (>-0.58)	0.77	0.94(0.64-1.40)	0.54	0.90(0.63-1.27)	0.45	0.89(0.66-1.20)
hsa_miR_7	Low(≤ -1.86) *vs.* High expression (> -1.86)	0.01	0.83(0.72-0.96)	0.0078	0.67(0.58-0.78)	<0.0001	0.49(0.39-0.62)
hsa_miR_769_5p	Low(≤ -3.95) *vs.* High expression (> -3.95)	0.19	0.67(0.37-1.22)	0.4	1.28(0.72-2.29)	0.41	0.94(0.83-1.08)
hsa_miR_92b	Low(≤ 0.44) *vs.* High expression (> 0.44)	0.73	1.05(0.81-1.35)	0.2	0.91(0.77-1.06)	0.54	0.94(0.76-1.15)
hsa_miR_93	Low(≤ -0.54) *vs.* High expression (> -0.54)	0.63	0.95(0.76-1.18)	0.51	1.07(0.88-1.29)	0.37	1.11(0.88-1.40)

**Figure 2:**
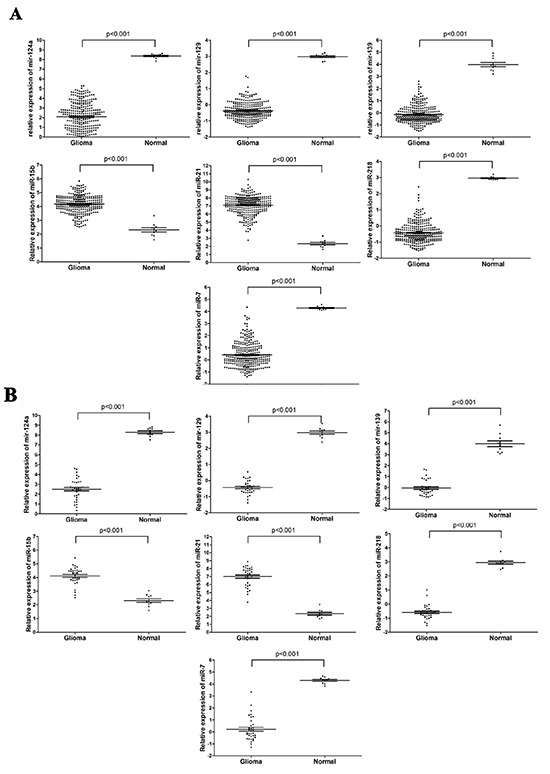
MiRNA expression profiles and quantitative RT-PCR validation of seven differentially expressed miRNAs **A.** The relative expression of miR-124a, miR-129, miR-139, miR-15b, miR-21, miR-218 and miR-7 from microarray analysis of 300 GBM samples and 10 healthy control samples from TCGA database. **B.** Relative expression of these same miRNAs was determined by quantitative RT-PCR of 34 GBM samples and 10 control non-cancerous glial tissues. Expression was normalized to U6 expression. Data are presented as the mean± SEM, and the statistical significance calculated using the unpaired t-test is indicated.

Quantitative RT-PCR was then used to validate the seven miRNAs for an additional set of 34 pathologically proven malignant glioma tissues and 10 non-cancer normal brain tissues from the Affiliated People's Hospital of Jiangsu University. The expression of these miRNAs was significantly different between malignant glioma and non-cancerous normal brain samples and was consistent with the microarray expression patterns (Figure 2).

### Establishment of a seven-miRNA-based signature that is associated with GBM disease risk

**Figure 3:**
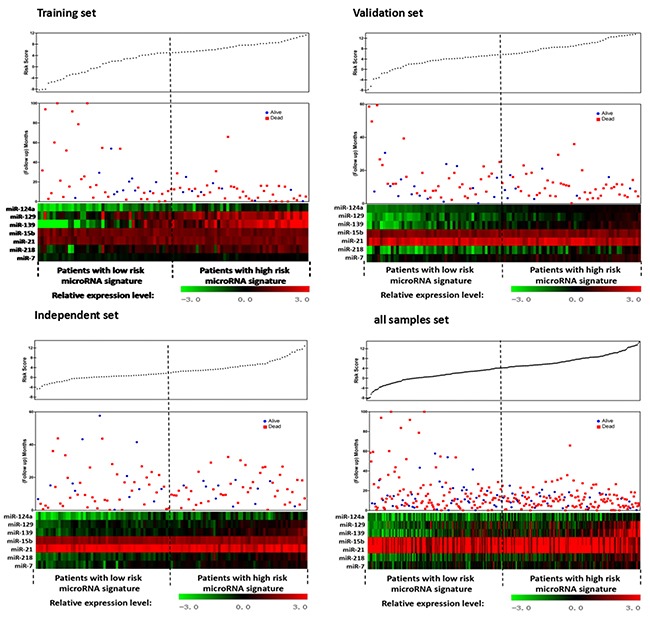
Seven-miRNA signature risk-score distribution in GBM patients from the training set, validation set and independent sets

**Figure 4:**
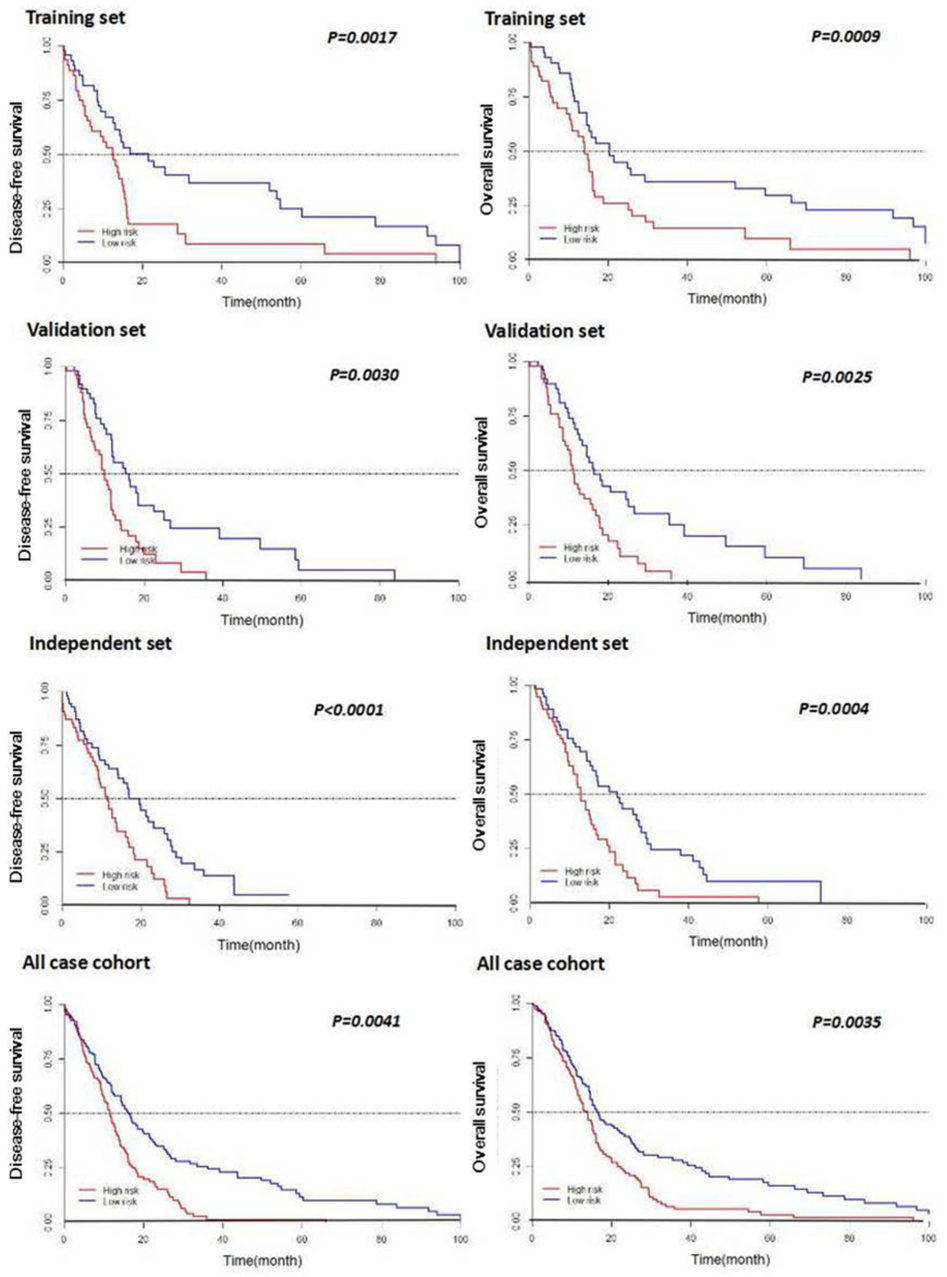
Kaplan-Meier curves of DFS and OS according to expression of the seven-miRNA signature in patients with GBM Correlations for patients with low-risk and high-risk miRNA expression scores are shown for the training set, the validation set, the independent set, and all patients combined. P-values were calculated by log rank testing.

To assess the utility of the seven miRNAs in predicting disease risk, we derived a formula using the LASSO Cox regression model to calculate a score for the risk of disease recurrence for patients based on their individual seven-miRNA expression levels. By introducing and observing auxiliary problems with continuous variables, a simple and efficient algorithm was established. Training set patients were further stratified into high- and low-risk groups, with the median risk score (4.25) as the cutoff value (Figure 3). No differences in clinical characteristics were observed between the two groups. However, Kaplan-Meier analysis showed that high-risk patients had shorter DFS and OS, which was confirmed for the three patient sets (Figure 4).

To further validate that these seven miRNAs are important for the signature, constructed six-miRNA signature was respectively created wherein one miRNA was sequentially deleted from the original seven-miRNA signature and survival analysis was compared by log-rank testing. Results showed that none of the iterations of the six-miRNA signatures was consistently correlated with DFS or OS in any of the three patient sets (Table 4).

**Table 4:** Log-rank test comparison of Kaplan-Meier survival analysis for the seven-miRNA signature vs. the “sevenminus- one” miRNA signatures in GBM patients

	Training set (109)	Validation set	Independent set
		Disease Free Survival	
7-microRNA signature	<0.0001	0.0066	0.0016
5-microRNA signature minus			
microRNA-124a	0.036	**0.124**	**0.059**
microRNA-129	**0.018**	0.0081	0.033
microRNA-139	0.0098	0.01	**0.058**
microRNA-21	0.025	0.0164	**0.083**
microRNA-218	0.027	**0.05**	0.0043
microRNA-15b	**0.072**	0.004	0.0012
microRNA-7	**0.08**	**0.147**	0.013
		**Overall Survival**	
7-microRNA signature	0.0014	0.0091	0.0035
5-microRNA signature minus			
microRNA-124a	0.042	**0.13**	0.042
microRNA-129	0.049	**0.171**	0.044
microRNA-139	0.006	**0.097**	**0.051**
microRNA-21	**0.09**	0.031	0.041
microRNA-218	0.04	**0.064**	0.0102
microRNA-15b	**0.12**	0.018	0.023
microRNA-7	**0.101**	**0.073**	0.037

### Comparison of the seven-miRNA-based signature to other parameters of disease risk

To compare the correlative ability of the seven-miRNA signature to that of other disease parameters, we performed univariate analysis. The results demonstrate that the risk score established by the miRNA expression was more effective at distinguishing DFS time than other criteria that are traditionally used to distinguish disease status, including Kamofsky score, tumor location, recurrence status, MGMT methylation, IDH1 mutation, smoking and family history of cancer. Similar results were obtained upon dividing the group into training and validation sets (Table 5).

**Table 5:** Univariate association of various parameters with DFS in the training, validation, and independent sets

Variables	Training set		Validation set		Independent set	
	p value	HR(95%CI)	p value	HR(95%CI)	p value	HR(95%CI)
Age (≤60 year vs.>60 year)	0.2	1.47(0.82-2.64)	0.67	0.88(1.69-5.42)	0.68	1.13(0.64-1.99)
Gender (Male vs. Female)	0.38	1.31(0.72-2.38)	0.66	0.89(0.49-1.58)	0.75	1.26(0.54-2.35)
Karnofsky score (≤70 score vs.≥80 score)	0.06	0.50(0.24-1.04)	0.66	1.13(0.52-1.51)	0.07	0.56(0.30-1.05)
Tumor location (Fronto-temporal vs. Other)	0.49	1.25(0.67-2.35)	0.61	0.87(0.65-1.98)	0.38	0.77(0.43-1.38)
Whether recurrence (Yes vs. No)	**0.02**	**0.44 (0.22-0.88)**	**0.03**	**1.88 (1.07-3.31)**	**0.023**	**0.49 (0.26-0.93)**
MGMT methylation (Yes vs. No)	0.49	0.80(0.43-1.50)	0.05	0.57(0.39-1.22)	0.7	1.11(0.64-1.94)
IDH1 mutant (Yes vs. No)	0.32	0.72(0.38-1.37)	0.25	0.68(0.32-0.99)	0.76	1.19(0.57-2.17)
Smoking (Yes vs. No)	0.22	0.65(0.32-1.29)	0.27	0.73(0.36-1.31)	0.56	1.21(0.63-2.33)
Family history of cancer (Yes vs. No)	0.88	0.94(0.45-1.98)	0.23	1.42(0.42-1.28)	0.3	1.42(0.73-2.79)
Seven-miRNA-based classifier (Low vs. High risk)	**<0.0001**	**3.55(1.82-6.94)**	**<0.0001**	**3.02(0.80-2.51)**	**0.0001**	**3.02(1.58-5.77)**

**Figure 5:**
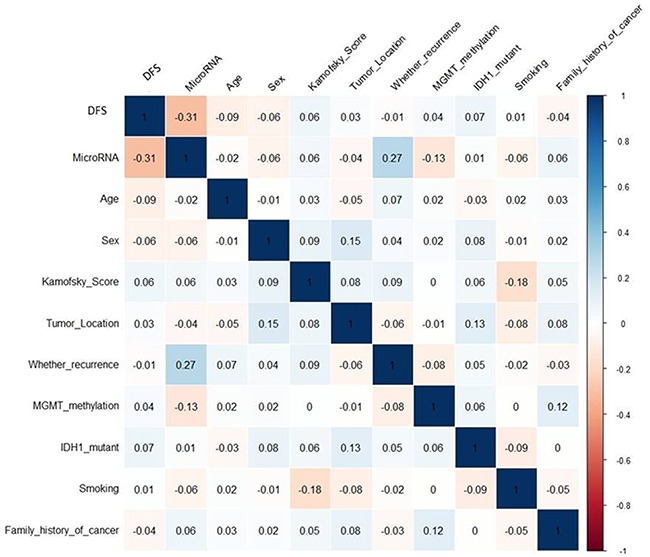
Correlation matrix of univariate association between miRNAs and other independent prognostic factors The matrix was generated by R language. The numbers in the squares indicate how the independent prognostic factors correlate with each other and seven-microRNA, the larger absolute value is the larger the correlation. Blue indicates positive correlation and read indicates negative correlation.

To further compare the seven-miRNA signature to other disease parameters, pairwise Pearson correlations were calculated for the signal values between 11 tracks (DFS, miRNA expression, age, sex, Kamofsky score, tumor location, recurrence, MGMT methylation, IDH1 mutation, smoking, and family history of cancer). The resulting matrix provides a pictorial representation showing that the miRNA signature has a high correlation with DFS and a high negative correlation with recurrence, whereas other parameters do not correlate as highly with DFS (Figure 5).

### Assessment of the prognostic capability of the seven-miRNA-based signature

**Figure 6:**
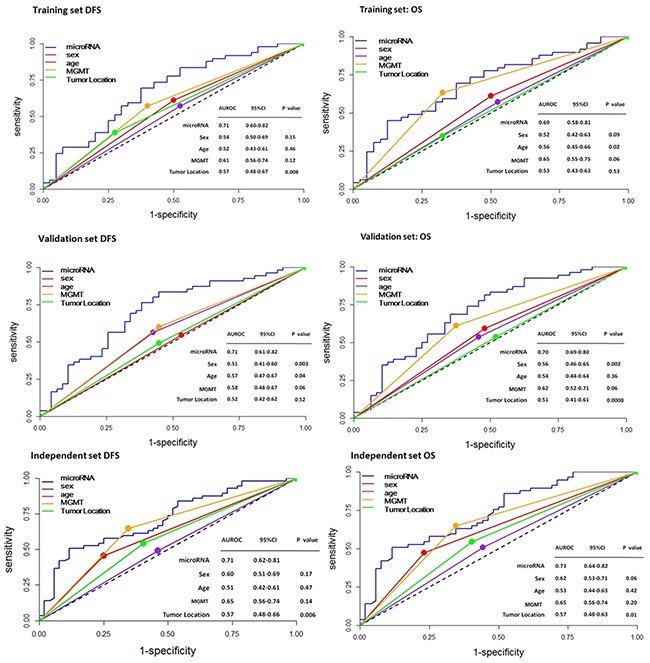
ROC analysis of the sensitivity and specificity for prediction of DFS and OS by the seven-miRNA signature, sex, age, MGMT, and tumor location in patients with GBM The values of the areas under the ROC curve (AUROC) are shown as a measure of the prognostic capability of each parameter.

To assess the prognostic capability of the seven-miRNA signature, we performed ROC analysis. The seven-miRNA signature provided a high sensitivity and specificity of survival prediction in the training set, validation set and independent set, with areas under the ROC curve (AUROC) ranging from 0.51 to 0.71 for DFS prediction and 0.69 to 0.73 for OS prediction (Figure 6).

**Figure 7:**
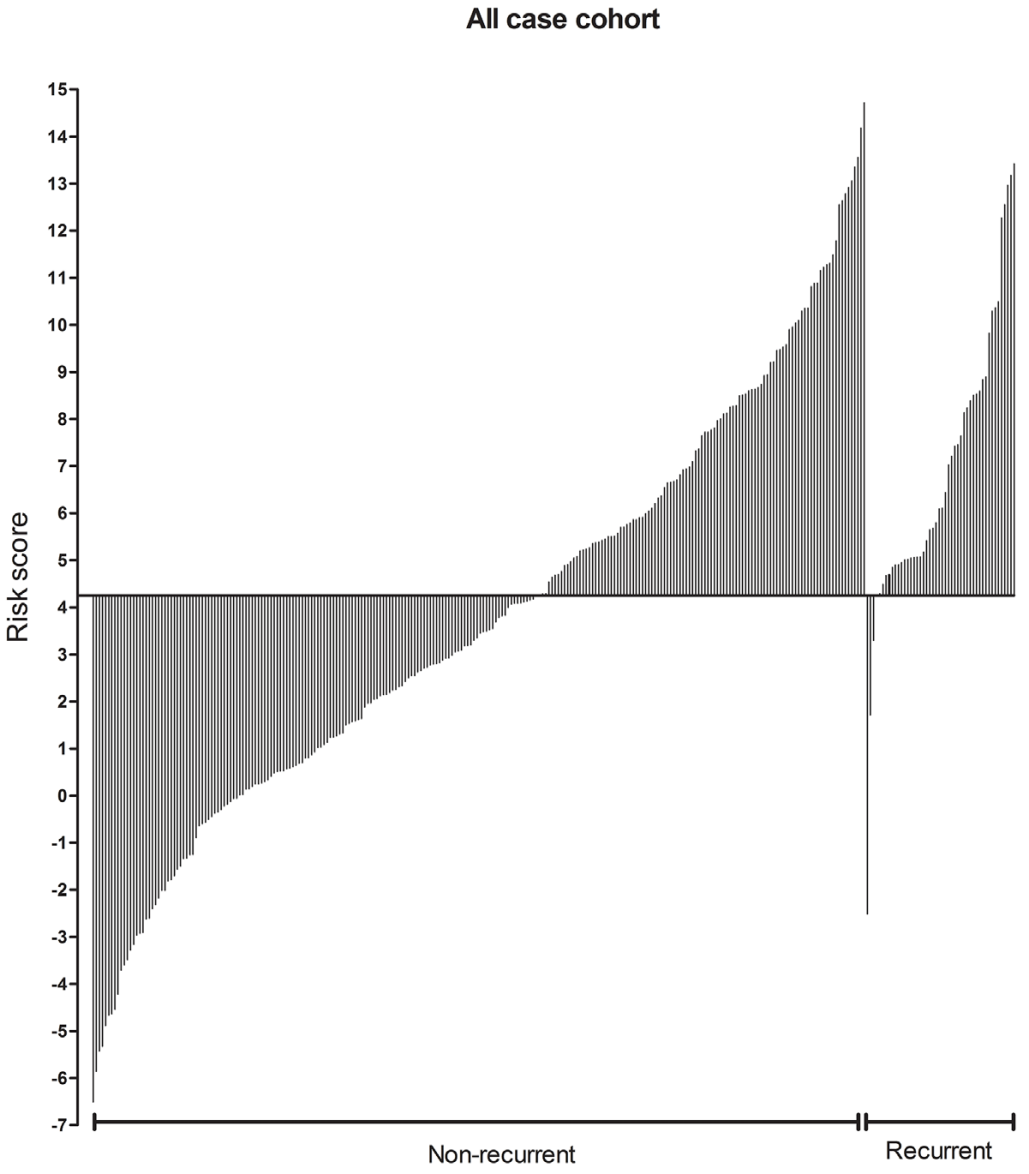
Stratification of patients on the basis of the seven-miRNA signature and recurrence The 300 GBM patients within this study were classified by the risk score defined in this study and divided into groups with and without recurrence in short times. The median time to recurrence from TCGA samples is 7 months.

To further examine the prognostic capability of the miRNA, we applied the same cutoff risk scores to all samples from TCGA in the cohort. A significant segregation of patients based on DFS (P < 0.001) was observed (Figure 7). These analyses underscore the robustness of our miRNA-based classifier in predicting glioma patient outcome.

### Analysis of the network of genes and pathways targeted by the 7-miRNA signature using Circos plots

**Figure 8:**
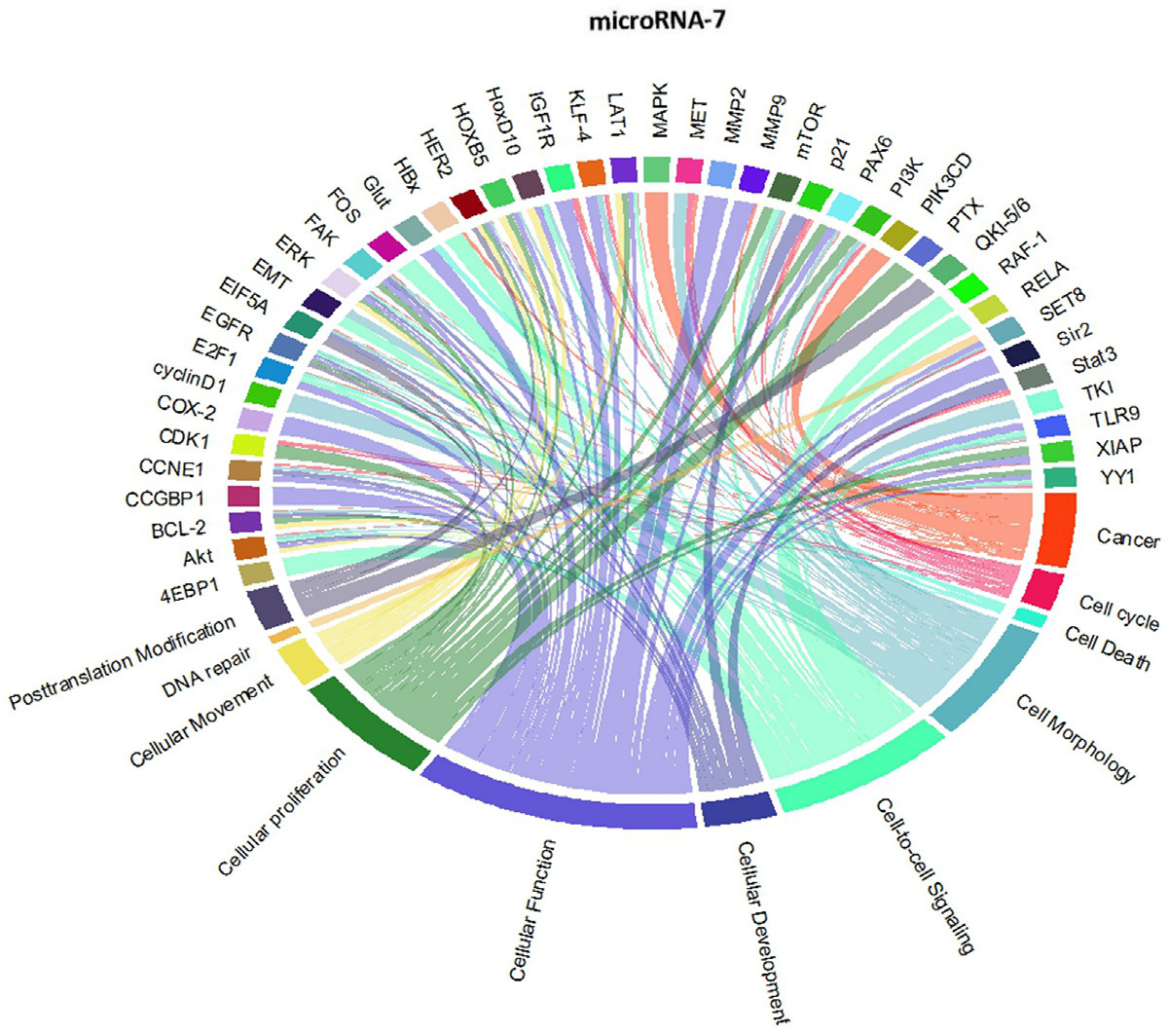
Circos plots of the metagenomic regulatory networks predicted to be targeted by the seven-miRNA classifier (miR-7, miR-15b, miR-21, miR-124a, miR-129, miR-139 and miR-218

**Figure 9:**
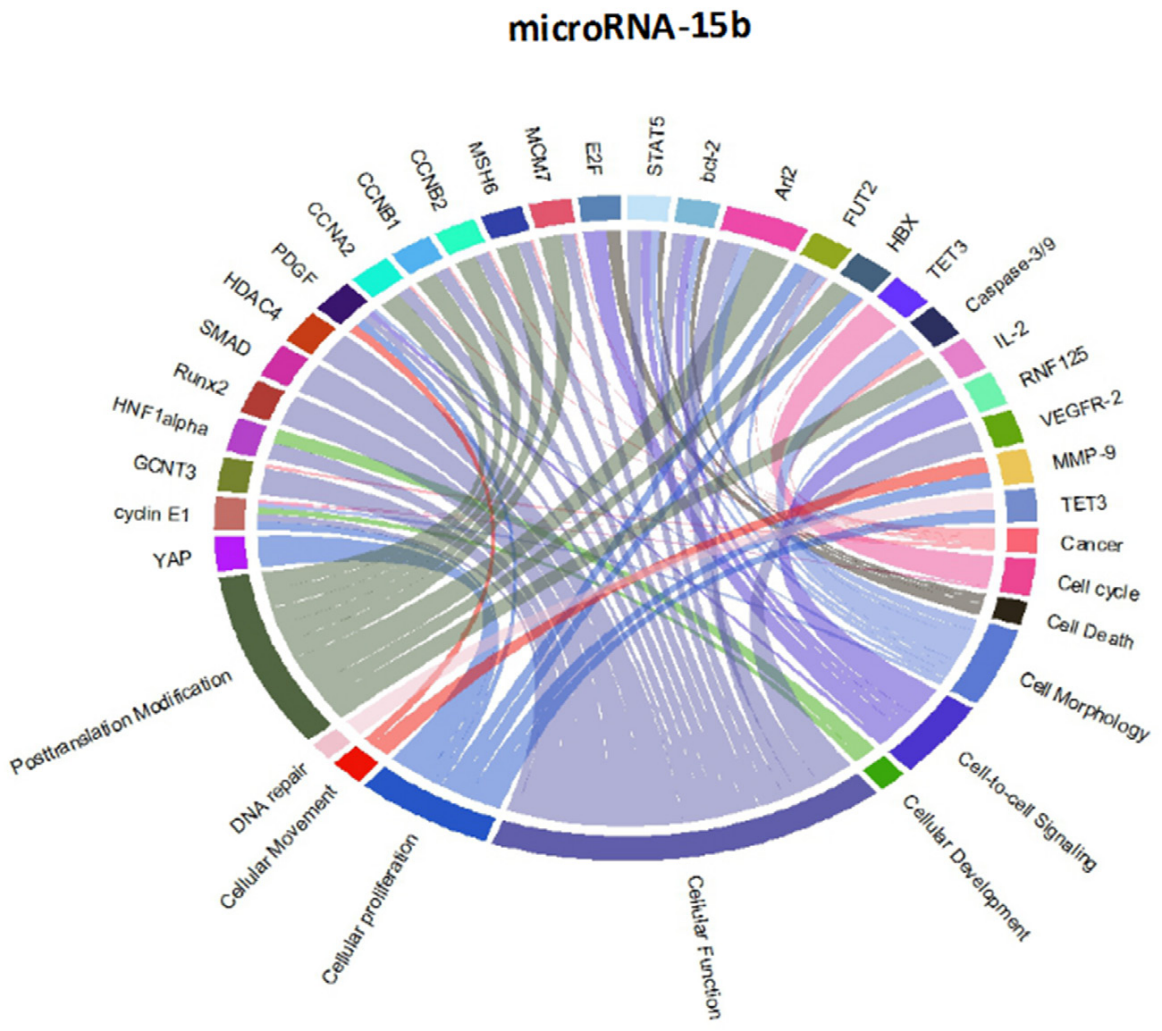
Circos plots of the metagenomic regulatory networks predicted to be targeted by the seven-miRNA classifier (miR-7, miR-15b, miR-21, miR-124a, miR-129, miR-139 and miR-218

**Figure 10:**
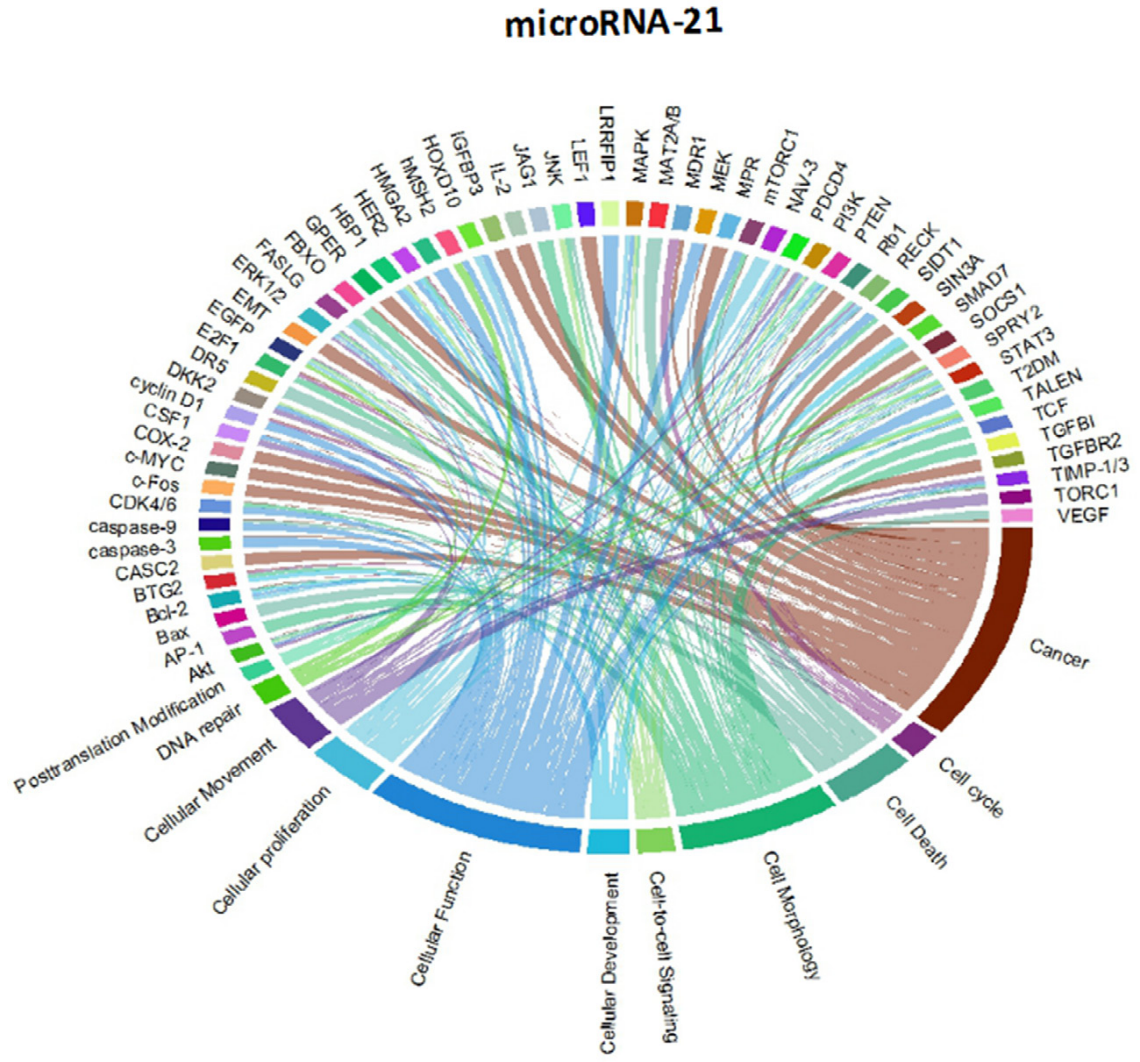
Circos plots of the metagenomic regulatory networks predicted to be targeted by the seven-miRNA classifier (miR-7, miR-15b, miR-21, miR-124a, miR-129, miR-139 and miR-218

**Figure 11:**
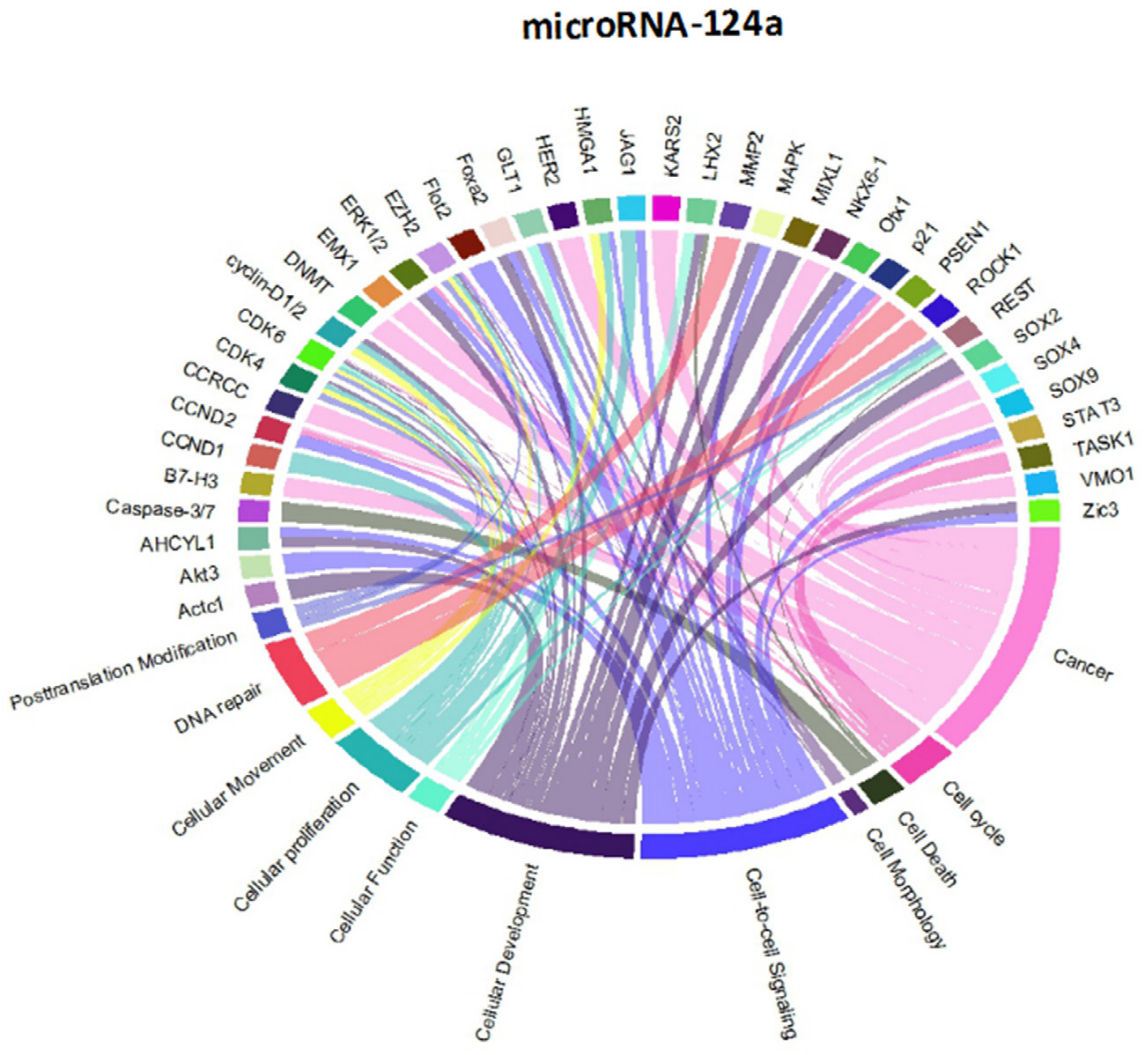
Circos plots of the metagenomic regulatory networks predicted to be targeted by the seven-miRNA classifier (miR-7, miR-15b, miR-21, miR-124a, miR-129, miR-139 and miR-218

**Figure 12:**
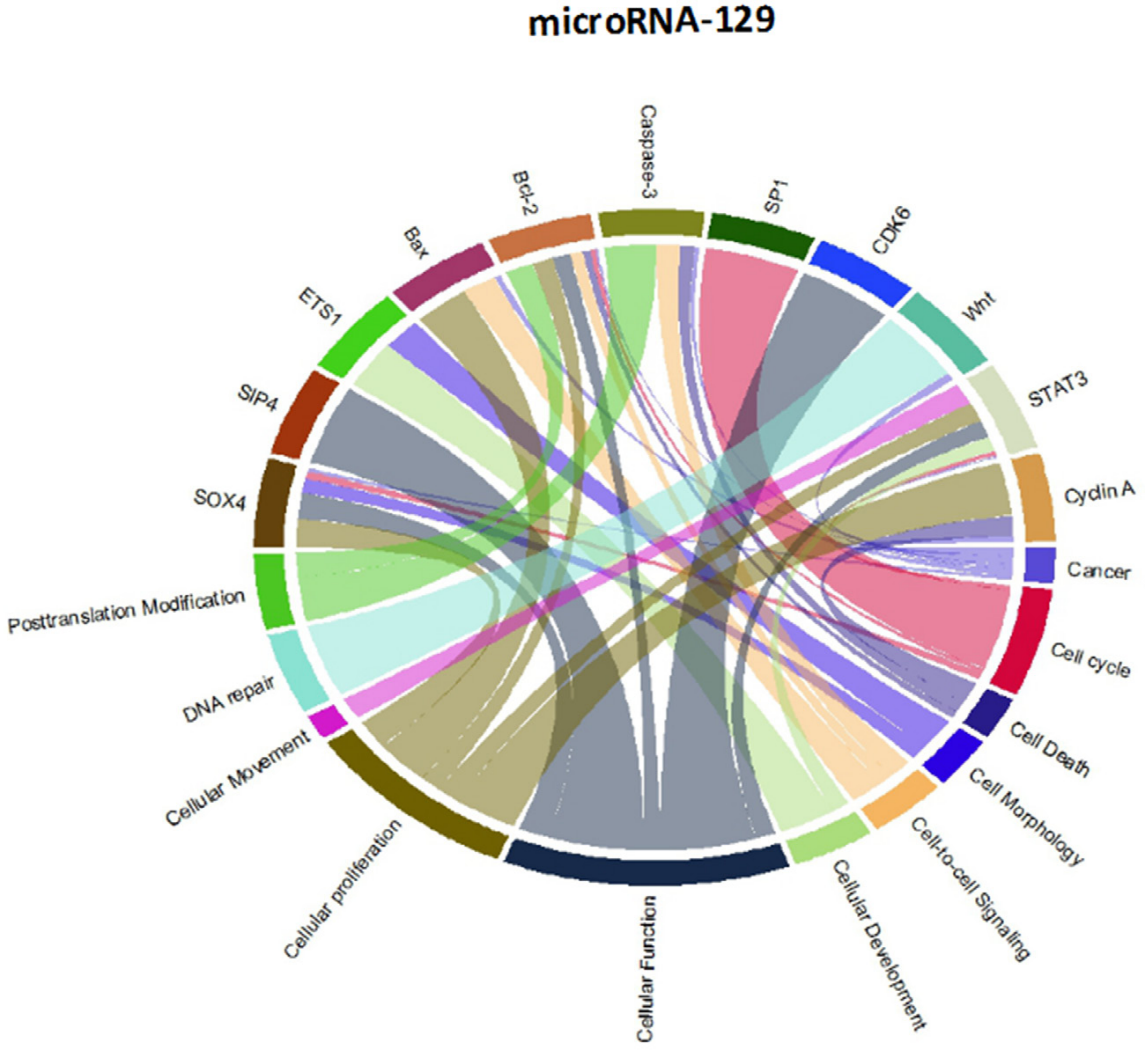
Circos plots of the metagenomic regulatory networks predicted to be targeted by the seven-miRNA classifier (miR-7, miR-15b, miR-21, miR-124a, miR-129, miR-139 and miR-218

**Figure 13:**
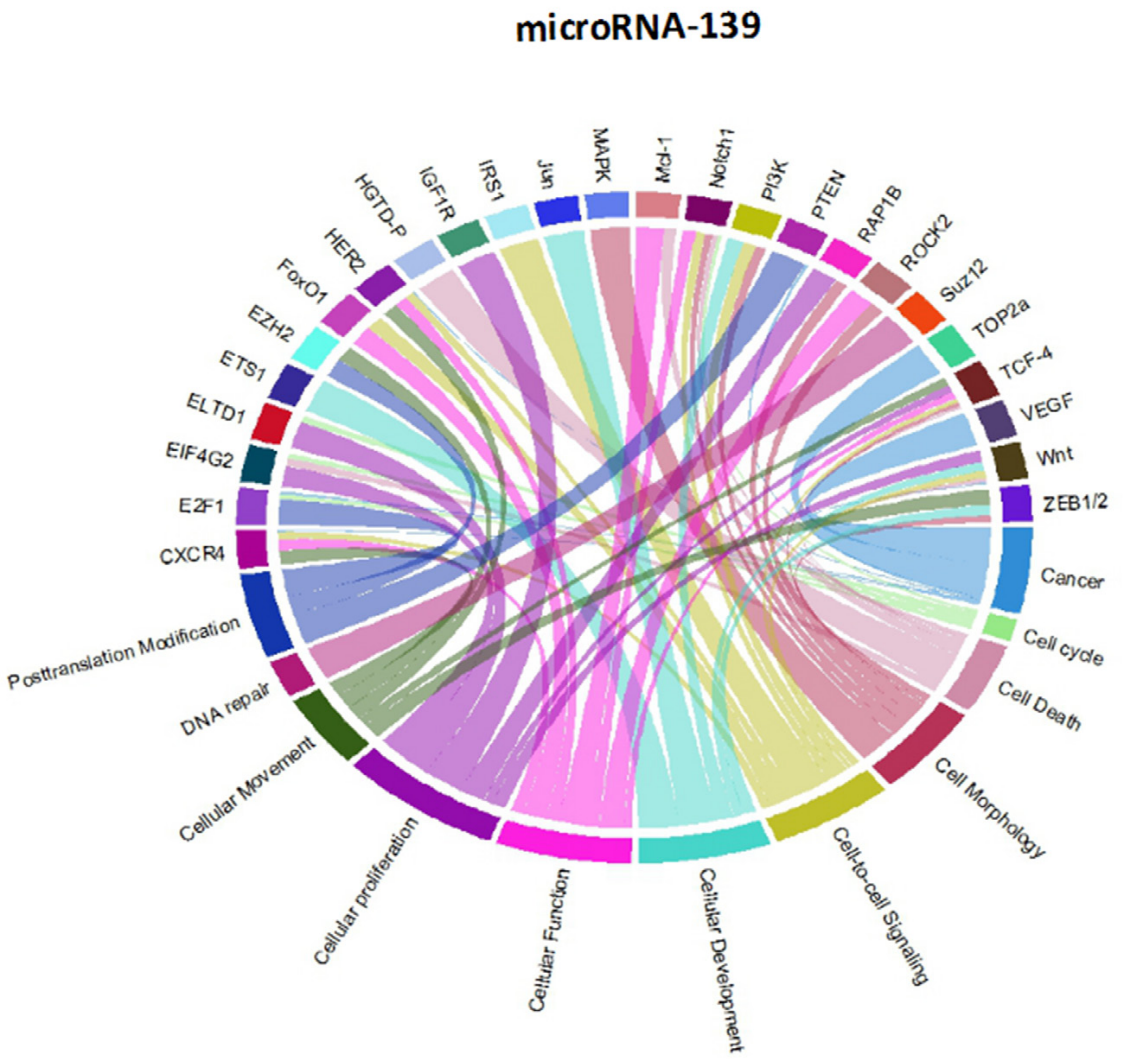
Circos plots of the metagenomic regulatory networks predicted to be targeted by the seven-miRNA classifier (miR-7, miR-15b, miR-21, miR-124a, miR-129, miR-139 and miR-218

**Figure 14:**
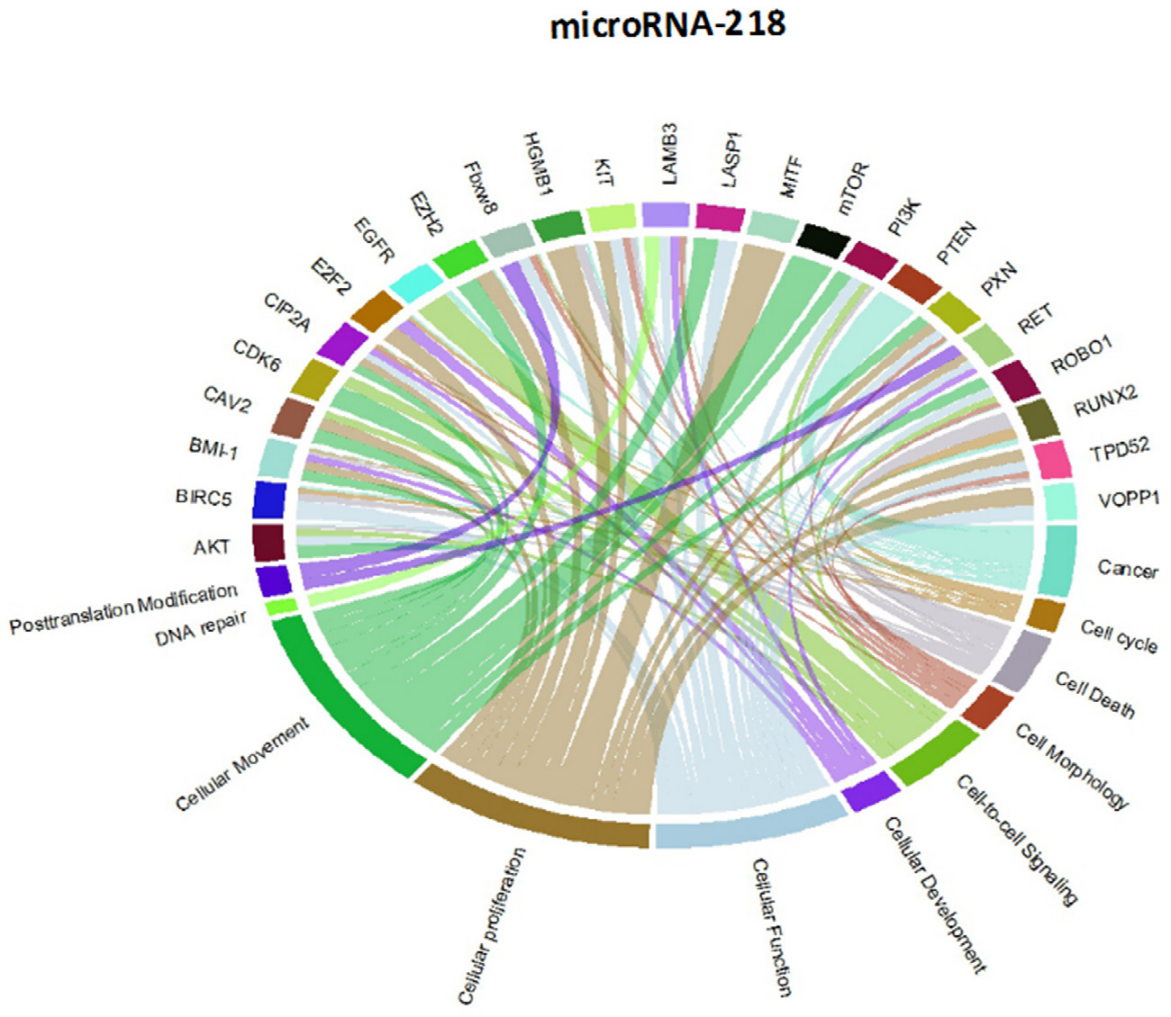
Circos plots of the metagenomic regulatory networks predicted to be targeted by the seven-miRNA classifier (miR-7, miR-15b, miR-21, miR-124a, miR-129, miR-139 and miR-218

Though initially designed for displaying comparative genomic data, Circos plots have also been used to analyze mutations in cancer metagenomic regulatory networks [18]. To provide a comprehensive evaluation of the putative biological functions of the seven-miRNA signature, we applied Circos plots to integrate data from the seven miRNAs, their predicted transcriptional targets, and annotated functions in glioma. To our knowledge, this is the first time that data from miRNAs and their predicted targets have been simultaneously combined in this manner. To decrease the complexity of the graphs and allow for better readability, we only showed selected transcriptional targets and inferred functions with known relevance to glioma. The results suggest that many oncogenes and tumor suppressor genes are targeted by miRNAs, including STAT3 and E2F1 (Figure 8-14).

## DISCUSSION

An excessive number of cancers occur without an effective means of prevention or treatment. In routine clinical practice, disease recurrence determines the prognosis of many glioma patients. However, the underlying molecular differences in glioma, even those with the same staging, leads to variation in patient outcomes, thus suggesting that there is a need for improvement of the current staging system [1]. To gain better insights into the biology of cancers, precision medicine has emerged as a promising approach for disease treatment and prevention that considers individual variability in genes, environment, and lifestyle [19]. Therefore, identifying novel genetic signatures may help clinicians to determine the likelihood of recurrence and tailor treatments accordingly.

In this study, we established a seven-miRNA diagnostic tool that can improve the predictability of disease recurrence. This novel prognostic tool was validated and our results showed that this tool can successfully stratify GBM patients in high- and low-risk categories and predict patient DFS. Additionally, in comparison with other clinicopathological risk factor analysis, the tool could more accurately predict patient survival, suggesting that the seven-miRNA-based classifier provides prognostic value that complements clinicopathological features.

Previous studies have identified multiple miRNAs that are differentially regulated in glioma compared with normal tissue. Each of the miRNAs in this study has previously been shown to be associated with prognosis or therapeutic outcome in patients with malignant glioma [7, 11-13, 20-25]; however, the integration of these miRNAs as a seven-miRNA signature for glioma prognosis is unique to our study. Interestingly, the two up-regulated miRNAs (miR-15b and miR-21) have been identified as circulating markers for glioma [24]. Furthermore, miR-15b has recently been identified as a “hub” gene that regulates multiple pathways associated with glioma progression [12]. Conversely, each of the down-regulated genes has been directly shown to have tumor suppressor properties, including the ability to inhibit glioblastoma invasion, migration and viability [13, 26-29]. These findings support the inclusion of the miRNAs within the seven-miRNA signature.

Our study is unique from previous studies in that it provides an approach for classifications of gliomas using a large sample size and advanced statistical approaches. Previous studies that have assessed miRNA biomarkers in glioma have been limited by small numbers of miRNAs screened, small sample sizes, lack of independent validation sets, and the use of inappropriate statistical methods to mine miRNA microarray data. The use of the LASSO Cox regression model has allowed us to integrate multiple miRNAs into one tool, which has significantly greater prognostic accuracy than that of single miRNAs alone [14]. We demonstrated that similarly-staged glioma patients can be classified into high- and low-risk groups based on their seven-miRNA signature identified by this method. Additionally, the seven-miRNA signature had a better survival prognostic capability compared with other indices as determined by ROC analysis. Thus, classification of patients by this approach could provide useful information to clinicians on the glioma recurrence risk and guide decisions for treatment. Based on the highly sensitive results of our prognostic model, modifications can be made on disease management, for example over-treatment of low-risk patients can be avoided which could save extraordinary expense and unnecessary aggravation.

Notably, we validated the differential expression of the seven miRNAs identified in this study using an independent set for formalin-fixed paraffin-embedded tissues. As we could not obtain fresh paired frozen specimens, a direct miRNA expression comparison between corresponding frozen and formalin-fixed paraffin-embedded clinical specimens could not be performed. However, studies on mouse liver show similar miRNA expression results for both freshly frozen and formalin-fixed paraffin-embedded specimens [30]; however, it still would be important to repeat the RT-PCR analyses of the seven-miRNA signature using fresh frozen samples. As an additional potential limitation, our study was performed using samples from the United States and China. The distribution of clinical characteristics could potentially differ in other geographic locations. Therefore, the generalizability of our results should be further validated on other prospective studies for population-based analysis.

In summary, our results suggest that the novel seven-miRNA based prognostic tool can effectively classify malignant glioma patients into groups at low or high risk of glioma relapse, thereby adding prognostic value to the traditional clinicopathological risk factors used to assess these patients' prognosis. Overall, we believe that the distinctive seven-miRNA classifier provides a useful prognostic tool with clinical value for appropriately categorizing patients with malignant glioma.

## MATERIALS AND METHODS

### Patients and glioma samples

Clinical information for 495 patients with GBM and 10 unmatched non-tumor samples from patients undergoing brain trauma were obtained from The Cancer Genome Atlas (TCGA) (tcga-data.nci.nih.gov”; accessed November 2013). The database includes both primary and secondary GBM. To rule out changes in miRNA expression caused by prior disease treatment, 308 patients that had not received radiotherapy of chemotherapy prior to biopsy sampling were extracted from the 495 in the database. We stochastically used computer-generated random numbers to select 300 of the 308 samples for analysis and to assign 89 of the samples to the training set, 102 samples to the validation set and the other 109 samples to the independent set. The demographic and clinical features of all patients and healthy controls are listed in Table 1.

In addition to the data from TCGA, we obtained 34 pathologically proven glioma (grade III–IV) specimens and 10 paraffin-embedded normal brain tissues from patients with brain trauma from the Affiliated People's Hospital of Jiangsu University between 2009 and 2015. The demographic and clinical features of these patients and healthy controls are shown in Table 6. All samples were assessed by pathologists of the Affiliated People's Hospital of Jiangsu University. The institutional review boards at each participating institution approved retrospective analysis of anonymous data.

**Table 6:** Baseline characteristics of patients in 34 GBM samples from the Affiliated People's Hospital of Jiangsu University

	Numbers of patients	Low risk	High risk
**Age**			
≤60 year	19	9(35%)	10(53%)
>60 year	15	4(27%)	11(73%)
**Gender**			
Male	19	8(420%)	11(58%)
Female	15	5(33%)	10(67%)
**Karnofsky score**			
≤70 score	22	7(32%)	15(68%)
≥80 score	12	6(50%)	6(50%)
**Tumorlocation**			
Fronto lobe or temporal lobe	12	5(42%)	7(58%)
Other lobe*	22	8(36%)	14(64%)
**Recurrence**			
Yes	14	2(14%)	12(86%)
No	20	11(55%)	9(45%)
**MGMT methylight**			
methylated	18	7(39%)	11(61%)
unmethylated	16	6(38%)	10(62%)
**IDH1 mutant**			
mutated	3	1(33%)	2(67%)
unmutated	31	12(39%)	19(61%)
**Smoking**			
Yes	11	3(27%)	8(73%)
No	23	10(43%)	13(57%)
**Family history of cancer†**			
Yes	18	4(22%)	14(78%)
No	16	9(56%)	7(44%)

* Other lobe including insular lobe,parietal lobe,cerebellar,lateral ventricles,fourth ventricle, occipital lobe and multiple lobes

### RNA Isolation, Microarray Analysis, and Quantitative RT-PCR

The isolation of total RNA from the 300 GBM samples and 10 unmatched non-tumor samples and microarray analysis has been described previously [16]. Data that were obtained using the Agilent human miRNA 8×15k microarray (level 3) containing 534 miRNA probes were downloaded from the 2013 TCGA repository. Level 3 data were derived after the raw signals per probe (level 1) were normalized per probe set (level 2) and then averaged for each miRNA. We performed a significance analysis of the microarray data, with a false discovery rate of less than 0.001, to identify miRNAs differentially expressed between the paired cancer and normal samples [17]. MiRNAs were classified as differentially expressed if the expression change was >3.0-fold, and the difference were considered to be significant if p-values were less than 0.01. We perform hierarchical clustering analysis with the average linkage method, and un-centered Pearson's correlation coefficients with MEV version 4.9.

RNA was isolated from the 34 glioma (grade III–IV) specimens and 10 normal brain tissues from the Affiliated People's Hospital of Jiangsu University by the Trizol method (Invitrogen, USA). RT-PCR was performed using the one-step SYBR RT-PCR Kit II (Takara^®^, Japan). Values were standardized to the expression of U6 RNA.

### Statistical analysis

The statistical software SPSS version 19.0 was used for data analysis. All computational work was performed using R language (v3.1.1). We analyzed data from TCGA for the selected miRNAs to evaluate their prognostic value in malignant glioma. The primary outcome of this paper was DFS. We assessed the relationship between the clinical characteristics and miRNA expression using the Student's *t* test, χ^2^ test or Fisher's exact test. We analyzed correlations between the microarray data and the RT-PCR data of our samples using the Spearman correlation test. We used the Kaplan-Meier method and the log-rank test to estimate DFS and OS, and calculated hazard ratios with an adjusted multivariate Cox regression analysis.

We used the LASSO Cox regression model [14] to select the most useful prognostic markers among the GBM-associated miRNAs identified with the cohort set, and we constructed a multi-miRNA-based classifier for predicting the DFS of patients with malignant glioma in the training set. We also used the R software version 3.1.1 “glmnet” package to perform the LASSO Cox regression model analysis. To identify the differentially expressed miRNAs that were significantly associated with DFS in the training set, we use LASSO Cox regarding analysis with R language. MiRNAs that were associated significantly with DFS were selected to construct an miRNA signature with the risk-score method and were used for survival analysis. The following algorithm was used to divide patients into high-risk (score > 4.25) and low-risk (score < 4.25) groups: risk score = (0.039 × status of miR-124a) + (0.389 × status of miR-129) + (0.495 × status of miR-139) - (0.396 × status of miR-15b) - (0.124 × status of miR-21) + (0.387 × status of miR-218) + (0.062 × status of miR-7). We compared the two groups using the *t* test for continuous variables and χ^2^ test for categorical variables.

For survival analyses, we used the Kaplan-Meier method to analyze the correlation between variables and DFS, and the log-rank test to compare survival curves. We used the Cox regression model to perform the multi-variable survival analysis, and Cox regression coefficients to generate nomograms. Calibration plots were generated to explore the performance characteristic of the nomograms. The x-axis represents the prediction calculated with use of the nomogram, and the y-axis represents the actual freedom from cancer recurrence for our patients. The 45-degree dashed line represents the performance of an ideal nomogram. Nomogram and calibration plots were generated with the rms using R software version 3.9.0. Statistical significance was set at 0.05.

We performed multivariate analysis using a backward stepwise approach to test if the signature was an independent prognostic factor of OS, age, gender, Karnofsky score, tumor location, recurrence, MGMT methylation, IDH1 mutation, smoking, or family history of cancer. Individual miRNAs within the 7-miRNA-based classifier were used as covariates. The observed two-tailed significance level was less than 0.001.

We also used R software version 3.1.1 and the “survival ROC” package to perform time-dependent receiver operating characteristic (ROC) curve analysis. We investigated the prognostic accuracy of each feature and multi-miRNA-based classifier using time-dependent ROC analysis. We assessed the area under the curve at different cutoff times to measure prognostic accuracy.

### Generation of a circos plot for identification of genes and pathways targeted by the seven-miRNA classifier

The Circos plots show glioblastoma signature genes regulated by miRNA. Several utility tools are bundled with Circos to help analyze, filter, and format data. Circos plots apply simulated annealing to a linked data set to generate an ideogram order that minimizes/maximizes the number of links that cross in the image. R language is a collection of tools that is used to parse tabular data and generate data and configuration files for visualizing tables with Circos.

## SUPPLEMENTARY FIGURES AND TABLES








