# Supplementary material for: The prognostic value of a seven-microRNA classifier as a novel biomarker for the prediction and detection of recurrence in glioma patients
Source: Oncotarget. 2016 Jul 11;7(33):53392–413. doi: 10.18632/oncotarget.10534 (PMC5288195; doi:10.18632/oncotarget.10534)
Supplement: Supplementary file 1 [file oncotarget-07-53392-s001.pdf]

The prognostic value of a seven-microRNA classifier as a novel biomarker for the prediction and detection of recurrence in glioma patients

SUPPLEMENTARY TABLES

Supplementary Table S1: MiRNAs differentially expressed in GBM tissues compared with non-cancerous glial tissues in the training set, validation set and independent set. The data were derived from 89, 102 and 109 GBM patients from TCGA database and 10 controls.

See Supplementary Table 1

Supplementary Table S2: MiRNAs differentially expressed in GBM tissues compared with non-cancerous glial tissues in the training set, validation set and independent set. The data were derived from 89, 102 and 109 GBM patients from TCGA database and 10 controls.

See Supplementary Table 2

Supplementary Table S3: MiRNAs differentially expressed in GBM tissues compared with non-cancerous glial tissues in the training set, validation set and independent set. The data were derived from 89, 102 and 109 GBM patients from TCGA database and 10 controls.

See Supplementary Table 3
